# Periodontal Disease and Salivary Gland Dysfunction in Neurofibromatosis Type 1: A Case–Control Study

**DOI:** 10.1111/odi.70306

**Published:** 2026-04-06

**Authors:** Eloá Borges Luna, Gustavo de Souza Vieira, Flávia Lima Kleinsorgen Motta, Ana Beatriz Oliveira Gambôa, Analúcia Rampazzo Xavier, Rafaela Elvira Rozza‐de‐Menezes, Karin Soares Cunha

**Affiliations:** ^1^ Graduate Program in Pathology, School of Medicine Universidade Federal Fluminense Niterói RJ Brazil; ^2^ Neurofibromatosis National Center (Centro Nacional de Neurofibromatose) Rio de Janeiro RJ Brazil; ^3^ Restorative Department Bristol Dental School and Hospital Bristol England UK; ^4^ Department of Pathology, School of Medicine Universidade Federal Fluminense Niterói RJ Brazil

**Keywords:** hyposalivation, neurofibromatosis 1, oral manifestations, periodontal diseases

## Abstract

**Objectives:**

Neurofibromatosis type 1 (NF1) presents with diverse systemic and oral manifestations. The aim of this study was to investigate the periodontal status and salivary alterations in NF1 individuals.

**Methods:**

A total of 38 individuals with NF1 diagnostic criteria were compared with a control group paired by age and sex. The periodontal indexes, sialometry at rest and under stimulation, saliva collection for biochemistry and pH evaluation, tongue coating, and oral hygiene indexes were performed. Socioeconomical and oral dryness questionnaire were also applied.

**Results:**

Individuals with NF1 had poor periodontal status according to the periodontal indexes compared to the control group (community periodontal index, *p* = 0.013; clinical attachment loss, *p* = 0.001; periodontal status, *p* = 0.002). Hyposalivation was significantly more prevalent in NF1 individuals at rest and under stimulation (*p* < 0.0001). There was also a high score on the dry mouth questionnaire in the NF1 group (evidencing xerostomia; *p* = 0.040), as well as greater and thicker tongue coating (*p* = 0.022). Salivary flow rates, pH, buffering capacity, biochemical composition, and socioeconomic levels did not differ between the periodontal classifications.

**Conclusion:**

Individuals with NF1 had poor periodontal status, higher rates of hyposalivation and oral dryness, and thicker and greater tongue coating than the control population.

## 1 Introduction

Neurofibromatosis 1 (NF1) is one of the most common genetic diseases in humans, with a prevalence of 1:3000 individuals (Riccardi 1992; Geller et al. 1998; Friedman et al. 1999). It results from pathogenic variants in the *NF1* gene, which encodes neurofibromin, a protein expressed in various cell types, including neurons, Schwann cells, osteoblasts, osteoclasts, and adrenal cells (Gutmann and Collins 1993; Tong et al. 2002). NF1 shows complete penetrance with variable expressivity (Riccardi 1992; Geller et al. 1998; Friedman et al. 1999).

The diagnosis of NF1 is established based on the revised 2021 criteria, which update the original NIH Consensus criteria from 1987 (NIH 1988; Legius et al. 2021). The most common manifestations include neurofibromas, café‐au‐lait spots, axillary and inguinal freckling, Lisch nodules in the iris, and choroidal abnormalities (NIH 1988; Riccardi 1992; Friedman et al. 1999; Legius et al. 2021). Oral alterations have been reported in over 90% of cases, affecting both soft and hard tissues of the stomatognathic system (Shapiro et al. 1984; D'Ambrosio et al. 1988; Cunha et al. 2004, 2015; Luna et al. 2018; Visnapuu et al. 2018; de Pinho Montovani et al. 2025).

In a previous study, our group reported a high prevalence of hyposalivation in individuals with NF1 (Cunha et al. 2015). Saliva plays a crucial role in oral homeostasis through antimicrobial properties and control of the microbial biofilm (Giannobile et al. 2009). Reduced salivary flow has been associated with increased susceptibility to oral infections, including periodontal diseases (Nederfors 2000; Amerongen and Veerman 2002; Turner and Sugiya 2002; Dawes 2008; Cunha et al. 2015; Dodds et al. 2015). In addition to flow rate, salivary biochemical composition—particularly calcium, magnesium, and phosphate concentrations—has been correlated with periodontal status in the general population (Sewón and Mäkelä 1990; Hernández‐Castañeda et al. 2015; Patel et al. 2016). High levels of these minerals may promote plaque calcification and periodontal inflammation (Sewón et al. 1988, 1998; Sewón and Mäkelä 1990; Schutzemberger et al. 2007; Fiyaz et al. 2013).

Periodontal diseases have a high impact on an individual's quality of life (Wong et al. 2021). The current classification and treatment guidelines for gingivitis and periodontitis focus on identifying risk factors for those diseases that can be addressed during step 1 of treatment (Chapple et al. 2018). Identifying unique risk factors relevant to NF1 individuals is clinically significant for effective treatment of periodontitis in this population.

Few studies have investigated periodontal diseases in NF1 individuals, and none have explored the relationship with salivary function, including flow rate and biochemical composition. Thus, this study aimed to investigate the periodontal status and salivary alterations in individuals with NF1 through a case–control approach.

## 2 Material and Methods

This observational, cross‐sectional, and case–control study was conducted at the Oral Diagnosis Service of the Antonio Pedro University Hospital (HUAP), affiliated with the Fluminense Federal University (UFF). Ethical committee approval was obtained under protocol number #1.645.631. Data collection and clinical examinations were conducted between 2016 and 2019.

The Study Group included 38 individuals with a diagnosis of NF1, and the Control Group comprised 38 individuals paired by sex and age. Diagnosis of NF1 was made based on the revised 2021 criteria (Legius et al. 2021). The control group was recruited from the Oral Diagnosis Service of the same hospital and geographic area as the NF1 group. The inclusion criteria were the absence of a diagnosis or family history of NF1 or any other monogenic genetic disorder. All participants underwent anamnesis, physical examination, sialometry, evaluation of the oral hygiene index and tongue coating, as well as periodontal examination. Exclusion criteria were: (1) Use of medications known to induce hyposalivation; (2) Current smoking; (3) Chronic alcohol consumption (> 10/15 doses [1 dose = 350 mL] per week) (Dutta et al. 1992); (4). Excessive intake of caffeinated beverages and/or stimulants (> 300 mL/day) (Hildebrandt et al. 2013); (5). History of systemic diseases that could affect salivary flow (diabetes, AIDS, autoimmune disease, endocrinopathies or neurological diseases); (6) History of head and neck oncologic treatment (chemotherapy or radiotherapy); (7) Complete edentulism. Participants also filled out a socioeconomic questionnaire, focusing on education level (based on the high school graduation) and average familial income for statistical analysis.

### 2.1 Periodontal Indexes Assessment

#### 2.1.1 Community Periodontal Index

The Community Periodontal Index (CPI) assessed periodontal status using probing pocket depth (PPD), bleeding on probing (BOP), and dental calculus presence, with a CPI probe. The mouth was divided into sextants (18–14, 13–23, 24–28, 38–34, 33–43, 44–48), and only sextants with at least two teeth without exodontia indication were examined. Each sextant was scored based on findings: (Code 0) healthy periodontium, (Code 1) gingival bleeding, (Code 2) supra‐ or sub‐gingival calculus, (Code 3) moderate periodontal pocket (3.5–5.5 mm), or (Code 4) deep periodontal pocket (≥ 5.5 mm).

#### 2.1.2 Clinical Attachment Loss

Clinical Attachment Loss (CAL) was assessed to determine the periodontal attachment status, measured as the distance from the cementoenamel junction (CEJ) to the base of the gingival sulcus or periodontal pocket. Measurements were categorized as < 4 mm (without CAL) or ≥ 4 mm (with CAL), according to published guidelines (Chapple et al. 2018).

#### 2.1.3 Periodontal Status

Individuals were classified according to the 2017 World Workshop on the Classification of Periodontal and Peri‐Implant Diseases and Conditions, based on their gingival and periodontal status as follows: (a) Clinical gingival health on an intact periodontium: absence of clinical signs of inflammation and no CAL; (b) Gingivitis on an intact periodontium: presence of gingival inflammation without CAL; (c) Gingivitis on a reduced periodontium: gingival inflammation at sites presenting attachment loss ≥ 4 mm (CAL); (d) Reduced periodontium without inflammation: sites with prior CAL due to periodontitis or other causes, with ≥ 4 mm attachment loss (CAL) and no BOP. Gingival health was defined as < 10% of sites with BOP. The extent of periodontal disease status was classified as localized and generalized when < 30% and ≥ 30% of teeth were affected, respectively (Papapanou et al. 2018; Chapple et al. 2018).

All periodontal examinations were performed by a single trained examiner with previous clinical experience in periodontal assessment. The standardized diagnostic criteria described above were strictly applied to ensure consistency and minimize measurement variability throughout the study.

### 2.2 Salivary Indicators Evaluation

#### 2.2.1 Oral Dryness Questionnaire

A specific seven‐question questionnaire previously published by our group (Cunha et al. 2015) was used to assess xerostomia and to analyze the clinical complaints corresponding to the symptoms of dry mouth. Each response was scored as 1 (never), 2 (occasionally), or 3 (always), and the total score was calculated for each participant.

#### 2.2.2 Sialometry

Sialometry was conducted in a quiet room in the morning (8:30–11:30 a.m.). Participants were instructed to avoid food, drinks, and tooth brushing at least 1 h before the test. The collection tube was placed near the lips, and participants let saliva drip passively into it with their mouths semi‐open. For the unstimulated whole saliva flow rate (UWSFR), they swallowed saliva and then drooled into the tube for 5 min without any stimulus. For the chewing‐stimulated whole saliva flow rate (CSWSFR), participants chewed a sterile sialagogue for 1 min, swallowed, then resumed chewing and drooling into the tube for 5 min. The saliva collected was measured with a 10 mL syringe, calculating flow rate in mL/min by measuring only the liquid component. Hyposalivation was defined as < 0.3 mL/min for UWSFR and < 0.7 mL/min for CSWSFR (Nederfors 2000). Samples were refrigerated for up to 4 h before biochemical analysis.

#### 2.2.3 Salivary pH and Buffering Capacity

For pH assessment, 0.2 mL of saliva was collected with a pipette, and the fluid was dripped onto MColorpHast pH indicator strips (Merck Chemical, Germany). To assess salivary buffer capacity (Moimaz et al. 2002), saliva was collected at rest and under stimulation. After rinsing with citric acid, the pH was measured, which was expected to be acidic. The participant then rinsed with water, waited 10 min, and saliva was collected again. If the pH returned to the initial stimulated value, buffer capacity was confirmed as preserved.

#### 2.2.4 Salivary Biochemistry

For salivary biochemical analysis, mineral (magnesium, potassium, phosphorus, and calcium) and enzymatic (amylase and lipase) components were evaluated. Mineral components were included due to their relevance to oral homeostasis, buffering capacity, mineral balance within the oral biofilm, and the oral microenvironment associated with periodontal tissue integrity, whereas enzymatic components were analyzed as indicators of salivary functional activity(Pedersen et al. 2018). Saliva samples collected at rest and/or under stimulation were immediately centrifuged at 10,000 rpm for 15 min at 4°C (SL‐5GR, Spinlab, BR). The supernatant was then aliquoted into 2 mL cryogenic tubes and stored at −80°C until analysis. Calcium, phosphorus, and magnesium levels were evaluated in a Cobas b 123 gasometer, metabolites and electrolytes (Roche Diagnostics, USA) using selective potentiometry/electrode; phosphorus and magnesium dosages with Kovalent colorimetric kits, using at least 800 μL of centrifuged saliva. To determine the appropriate dosage of salivary amylase and salivary lipase, a correction calculation was performed by normalizing the initial enzyme dosage to the total salivary protein content measured under resting and stimulated conditions. The absolute concentration of each salivary component was considered for statistical analysis.

### 2.3 Assessing Other Oral Health Indexes

The Simplified Oral Hygiene Index (S‐OHI) by Greene and Vermillion (Greene and Vermillion 1964) was used to assess oral hygiene, focusing on biofilm and dental calculus. After isolating the teeth with cotton rolls, biofilm was stained with a dye solution (Eviplac, Biodynamic, Brazil) and evaluated on a scale from 0 to 3, indicating the extent of biofilm and calculus coverage (0 = none, 1 = up to 1/3, 2 = up to 2/3, 3 = more than 2/3 of the surface). Tongue coating was visually quantified from tongue dorsum photographs, divided into nine sections, and rated from 0 to 2 (0 = no coating, 1 = fine, 2 = thick) (Shimizu et al. 2007). The sum was divided by 18, multiplied by 100, yielding a percentage index, with values over 50% indicating significant tongue coating.

### 2.4 Statistical Analysis

Data analysis was performed using SPSS software version 20.0. Continuous variables were compared using paired *t*‐test or Wilcoxon‐signed rank test, Student's *t*‐test or Mann–Whitney, and Kruskal‐Wallis or One‐Way ANOVA, based on normality and homogeneity (Shapiro–Wilk and Levene's tests). Fisher's exact or Pearson Chi‐square tests compared categorical variable frequencies. The McNemar Chi‐square or McNemar‐Bowker test assessed paired proportions by periodontal status between NF1 and control groups. Odds ratios (OR) with 95% confidence intervals (CI) estimated explanatory variables, and Spearman's rank correlation was used to assess correlations between continuous variables. Qualitative data were presented as percentages; *p*‐values ≤ 0.05 indicated significance.

## 3 Results

### 3.1 Sample Characteristics

The study included 38 individuals with neurofibromatosis type 1 (NF1) and 38 age‐ and sex‐matched controls. Baseline demographic and socioeconomic characteristics are presented in Table 1.

**TABLE 1:** Baseline demographic and socioeconomic characteristics of NF1 individuals and matched controls.

Variable	NF1 group (*n* = 38)	Control group (*n* = 38)
Age (years), mean ± SD	37.74 (±14.52)	37.74 (±14.52)
Sex, *n* (%)		
Female	28 (73.7%)	28 (73.7%)
Male	10 (26.3%)	10 (26.3%)
Educational level, *n* (%)		
High school completed	30 (79%)	24 (63.2%)
Higher education	3 (7.9%)	14 (36.8%)
Familial income (minimum wages), mean ± SD	4.16 (±3.79)	4.66 (±2.43)

*Note:* Groups were matched by age and sex. Smoking status, alcohol consumption, systemic diseases, medication use, history of head and neck oncologic treatment, and complete edentulism were defined as exclusion criteria and applied equally to both groups.

### 3.2 Periodontal Index Comparison

No participants were classified as CPI codes 2, 3, or 4 (supra or subgingival calculus, moderate periodontal pockets of 3.5–5.5 mm, or deep periodontal pockets ≥ 5.5 mm); therefore, only CPI codes 0 (healthy periodontium) and 1 (gingival bleeding) were included in the analysis. CPI code 1 participants accounted for 39.5% of the NF1 group and 10.5% of the control group. Presence of CAL ≥ 4 mm was observed in 68.4% and 28.9% of NF1 and control patients, respectively. The prevalence was nearly six‐fold higher in NF1 individuals in code 1 compared with those in code 0: 36.8% versus 7.9%. Therefore, there was a statistically significant difference in the CPI and CAL indexes (McNemar chi‐square test; *p* = 0.013 and *p* = 0.001, respectively) matching NF1‐control groups, as shown in Table 2.

**TABLE 2:** Cross‐tabulation of periodontal indexes and salivary flow classification in individuals with neurofibromatosis type 1 and sex and age‐matched controls.

Community periodontal index					
		NF1 Group			*p* ^a^
Controls		Code 1	Code 0	Total	0.013
	Code 1	1 (2.6%) 0.6^b^	3 (7.9%) 0.6^b^	4 (10.5%)	
	Code 0	14 (36.8%) 0.6^b^	20 (52.6%) 0.6^b^	34 (89.5%)	
	Total	15 (39.5%)	23 (60.5%)	38 (100%)	

Clinical attachment loss					
		NF1 Group			*p* ^a^
Controls		With CAL	Without CAL	Total	0.001
	With CAL	8 (21.1%) 0.4^b^	3 (7.9%) 0.4^b^	11 (28.9%)	
	Without CAL	18 (47.4%) 0.4^b^	9 (23.7%) 0.4^b^	27 (71.1%)	
	Total	26 (68.4%)	12 (31.6%)	38 (100%)	

Periodontal status							
		NF1 Group					*p* ^c^
		CGHIP	GIP	GRP	RP	Total	0.002
	CGHIP	8 (21.1%) 0.9^b^	0 (0%) 2.1^b^	8 (21.1%) 0.7^b^	10 (68.4%) 0.8^b^	26 (68.4%)	
Controls	GIP	0 (0%) 0.6^b^	1 (2.6%) 4.3^b^	0 (0%) 0.7^b^	0 (0%) 0.7^b^	1 (2.6%)	
	GRP	2 (5.3%) 1.7^b^	0 (0%) 0.4^b^	0 (0%) 1.3^b^	1 (2.6%) 0^b^	3 (7.9%)	
	RP	0 (0%) 1.9^b^	1 (12.5%) 1.0^b^	5 (13.2%) 1.9^b^	2 (5.3%) 0.6^b^	8 (21.1%)	
	Total	10 (26.3%)	2 (5.3%)	13 (34.2%)	13 (34.2%)	38 (100%)	

Salivary flow at rest					
		NF1 Group			*p* ^a^
Controls		HYP (+)	HYP (−)	Total	< 0.001
	HYP (+)	0 (−) 1.4^b^	1 (2.6%) 1.4^b^	1 (2.6%)	
	HYP (−)	25 (65.8%) 1.4^b^	12 (31.6%) 1.4^b^	37 (97.4%)	
	Total	25 (65.8%)	13 (34.2%)	38 (100%)	

Salivary flow under stimulation					
		NF1 Group			*p* ^d^
Control		HYP (+)	HYP (−)	Total	—
	HYP (+)	0 (−)	0 (−)	0 (−)	
	HYP (−)	15 (39.5%)	23 (60.5%)	38 (100%)	
	Total	15 (39.5%)	23 (60.5%)	38 (100%)	

Abbreviations: CAL, clinical attachment loss; CGHIP, clinical gingival health on an intact periodontium; CPI code 0, healthy periodontium; CPI code 1, gingival bleeding; CPI, community periodontal index; GB, gingival bleeding; GIP, gingivitis on an intact periodontium; GRP, gingivitis on a reduced periodontium; HP, health periodontium; HYP (−), number of individuals with normal salivary flow; HYP (+), number of individuals with hyposalivation; NF1, neurofibromatosis type 1; RP, reduced periodontium.

^a^ McNemar Chi‐square test.

^b^ Adjusted residuals.

^c^ McNemar‐Bowker Test.

^d^ *p*‐value not applicable because no controls presented hyposalivation for comparison.

In view of the periodontal status, clinical gingival health was significantly lower (26.3% vs. 68.4%), while gingivitis on a reduced periodontium (34.2% vs. 7.9%) and loss of attachment without gingivitis (34.2% vs. 21.1%) were higher in the NF1 group. Gingivitis on an intact periodontium was rare in both groups but slightly more frequent in the NF1 individuals (5.3% vs. 2.6%; McNemar‐Bowker test; *p* = 0.002; Table 2).

For molars and premolars, the proportion of sextants classified as CPI code 0 was significantly lower in the NF1 group (54.8%) than in controls (81%; McNemar chi‐square test; *p* = 0.013), whereas CPI code 1 was more frequent in the NF1 group (35.7% vs. 9.5%). In addition, clinical attachment loss ≥ 4 mm in posterior regions was significantly higher in the NF1 group (57.1%) compared with controls (23.8%; McNemar test; *p* = 0.001; Table 3). Generalized gingivitis on a reduced periodontium was more prevalent in the NF1 group (28.9%) than in controls (5.3%). Similarly, localized and generalized reduced periodontium were also more frequent among individuals with NF1 (*p* = 0.019; McNemar‐Bowker test; Table 3).

**TABLE 3:** Regional distribution of periodontal indexes in individuals with neurofibromatosis type 1 and matched‐sex and age controls.

		Sextants 1,3,4,6 (molars/premolars)			Sextants 2,5 (incisives/canines)		
		Controls (*n* = 38)	NF1 (*n* = 38)	*p*	Controls (*n* = 38)	NF1 (*n* = 37)	*p*
Community periodontal index^c^	Code 0	34 (81%)	23 (54.8%)	0.013^a^	35 (83.3%)	29 (69%)	0.227^a^
	Code 1	4 (9.5%)	15 (35.7%)		3 (7.1%)	8 (19%)	
Clinical attachment loss	Without CAL (< 4 mm)	28 (66.7%)	14 (33.3%)	0.001^a^	34 (81%)	23 (54.8%)	0.021^a^
	With CAL (≥ 4 mm)	10 (23.8%)	24 (57.1%)		4 (9.5%)	14 (33.3%)	

		Regional distribution of periodontal disease		
		Control (*n* = 38)	NF1 (*n* = 38)	*p*
Periodontal status	CGHIP	26 (68.4%)	10 (26.3%)	0.019^b^
	Localized GIP	0 (0%)	0 (0%)	
	Generalized GIP	1 (2.6%)	2 (5.3%)	
	Localized GRP	1 (2.6%)	2 (5.3%)	
	Generalized GRP	2 (5.3%)	11 (28.9%)	
	Localized RP	2 (5.3%)	8 (21.1%)	
	Generalized RP	6 (15.8%)	5 (13.2%)	

Abbreviations: CAL, clinical attachment loss; CGHIP, clinical gingival health on an intact periodontium; CPI code 0, healthy periodontium; CPI code 1, gingival bleeding; CPI, community periodontal index; GIP, gingivitis on an intact periodontium; GRP, gingivitis on a reduced periodontium; NF1, neurofibromatosis type 1; RP, reduced periodontium.

^a^ McNemar chi‐square test.

^b^ McNemar‐Bowker test.

^c^ No patients with codes 2, 3, and 4.

### 3.3 Salivary Index Comparison

Hyposalivation was significantly more prevalent in the NF1 group at rest (65.8% vs. 2.6%; McNemar chi‐square test, *p* < 0.0001) and under stimulation (39.5% vs. 0%; Table 2). Resting salivary flow was significantly reduced in NF1 individuals (0.29 ± 0.19 mL/min) compared to controls (0.70 ± 0.28 mL/min; Wilcoxon signed‐rank test; *p* < 0.001), as was stimulated salivary flow (NF1: 0.82 ± 0.33 mL/min; controls: 1.21 ± 0.31 mL/min; Wilcoxon signed‐rank test; *p* < 0.001; Table 4).

**TABLE 4:** Distribution of oral and salivary variables in the general population and according to the periodontal indexes in the neurofibromatosis type 1 group.

	*n*	General individuals			Community periodontal index			Clinical atachment loss			Periodontal status				
		Controls Mean (±SD)	NF1 Mean (±SD)	*p*	Code 0 Mean (±SD)	Code 1 Mean (±SD)	*p*	Without CAL Mean (±SD)	With CAL Mean (±SD)	*p*	CGHIP Mean (±SD)	GIP Mean (±SD)	GRP Mean (±SD)	RP Mean (±SD)	*p*
Oral hygiene index	36	0.67 (±0.48)	0.74 (±0.51)	0.620^a^	0.67 (±0.48) (*n* = 23)	0.94 (±0.50)	0.130^d^	0.70 (±0.44) (*n* = 12)	0.82 (±0.52) (*n* = 26)	0.540^d^	0.62 (±0.49) (*n* = 9)	0.61 (±0.72) (*n* = 2)	1.01 (±0.49) (*n* = 11)	0.65 (±0.53) (*n* = 12)	0.035^e^
Tongue coating	37	32.6% (±22.76)	43.0% (±21.96)	0.020^a^	33.64% (±18.57) (*n* = 22)	56.80% (±19.52) (*n* = 15)	0.001^c^	30.09% (±19.80) (*n* = 11)	48.50% (±20.80) (*n* = 26)	0.022^c^	26.33% (±15.70) (*n* = 9)	47.00% (±35.35) (*n* = 2)	58.31% (±17.95) (*n* = 13)	38.69% (±19.27) (*n* = 13)	0.007^e^
Oral dryness questionnaire (final score)	38	10.81 (±3.18)	12.39 (±3.10)	0.040^a^	13.43 (±3.02) (*n* = 23)	10.80 (±2.56) (*n* = 15)	0.005^c^	12.30 (±3.25) (*n* = 12)	12.58 (±2.87) (*n* = 26)	0.660^c^	13.10 (±2.84) (*n* = 10)	10.00 (±1.41) (*n* = 2)	10.92 (±2.72) (*n* = 13)	13.69 (±3.25) (*n* = 13)	0.040^e^
Salivary flow at rest (mL/min)	38	0.70 (±0.28)	0.29 (±0.19)	< 0.001^b^	0.29 (±0.21) (*n* = 23)	0.30 (±0.17) (*n* = 15)	0.820^c^	0.31 (±0.20) (*n* = 12)	0.29 (±0.19) (*n* = 26)	0.570^c^	0.3 (±0.22) (*n* = 10)	0.25 (±0.07) (*n* = 2)	0.3 (±0.18) (*n* = 13)	0.27 (±0.21) (*n* = 13)	0.929^e^
Salivary flow under stimulation (mL/min)	38	1.21 (±0.31)	0.82 (±0.33)	< 0.001^b^	0.82 (±0.31) (*n* = 23)	0.82 (±0.38) (*n* = 15)	0.890^c^	0.74 (±0.25) (*n* = 12)	0.85 (±0.36) (*n* = 26)	0.410^c^	0.7 (±0.25) (*n* = 10)	0.95 (±0.07) (*n* = 2)	0.8 (±0.4) (*n* = 13)	0.91 (±0.33) (*n* = 13)	0.536^e^
Salivary pH at rest	35	6.8 (± 0.57)	6.8 (±0.55)	0.570^a^	6.8 (±0.52) (*n* = 20)	6.9 (± 0.59) (*n* = 15)	0.500^c^	6.8 (±0.42) (*n* = 10)	6.8 (±0.60) (*n* = 25)	0.750^c^	6.8 (±0.35) (*n* = 8)	6.5 (±0.7) (*n* = 2)	7.0 (±0.57) (*n* = 13)	6.75 (±0.62) (*n* = 12)	0.658^e^
Salivary pH under stimulation	38	7.5 (±0.54)	7.5 (±0.54)	1.00^a^	7.60 (±0.49) (*n* = 23)	7.53 (± 0.63) (*n* = 15)	0.83^c^	7.41 (±0.66) (*n* = 12)	7.65 (±0.48) (*n* = 26)	0.29^c^	7.6 (±0.51) (*n* = 10)	6.5 (±0.7) (*n* = 2)	7.6 (0.48) (*n* = 13)	7.61 (±0.5) (*n* = 13)	0.535^e^
Salivary buffering capacity^f^—Acidic pH	32	3.68 (±0.69)	3.59 (±0.66)	0.263	3.54 (±0.67) (*n* = 22)	3.70 (± 0.67) (*n* = 10)	0.840^c^	3.55 (±0.88) (*n* = 9)	3.60 (±0.58) (*n* = 23)	0.58^c^	3.55 (±0.88) (*n* = 9)	—	3.70 (± 0.67) (*n* = 10)	3.53 (±0.51) (*n* = 13)	0.875^e^
Salivary buffering capacity^f^—Final pH	32	7.2 (±0.41)	7.2 (±0.41)	1.000^a^	7.22 (±0.42) (*n* = 22)	7.20 (±0.42) (*n* = 10)	0.920^c^	7.22 (±0.44) (*n* = 9)	7.21 (±0.42) (*n* = 23)	0.970^c^	7.2 (±0.44) (*n* = 9)	—	7.2 (±0.42) (*n* = 10)	7.23 (±0.43) (*n* = 13)	0.953^e^

Abbreviations: CAL, clinical attachment loss; CGHIP, clinical gingival health on an intact periodontium; CPI code 0, healthy periodontium; CPI code 1, gingival bleeding; CPI, community periodontal index; GIP, gingivitis on an intact periodontium; GRP, gingivitis on a reduced periodontium; NF1, neurofibromatosis type 1; RP, reduced periodontium.

^a^ Paired *T*‐Test.

^b^ Wilcoxon signed ranks test.

^c^ Mann–Whitney test.

^d^ *t* student test.

^e^ Kruskall–Wallis test.

^f^ Assessed after acid challeging, as described in Methods.

Xerostomia‐related symptoms, assessed via questionnaire, were significantly more frequent in the NF1 group (*p* = 0.040; paired *t*‐test). Due to insufficient salivary volume in some samples, pH and buffering capacity analyses at rest were performed to 38 participants or fewer, depending on the parameter. No significant differences in pH or buffering capacity were observed at rest or under stimulation (Table 4).

Salivary biochemical analysis revealed no significant intergroup differences in concentrations of calcium, magnesium, phosphorus, potassium, amylase, or lipase in either resting or stimulated saliva (Table 5).

**TABLE 5:** Salivary biochemical parameters at rest and under stimulation according to periodontal status in individuals with Neurofibromatosis type 1.

			Calcium		Magnesium		Phosphorous		Potassium		Amylase		Lipase	
		*n*	Mean (±SD)	*p*	Mean (±SD)	*p*	Mean (±SD)	*p*	Mean (±SD)	*p*	Mean (±SD)	*p*	Mean (±SD)	*p*
All individuals	UWSFR	31	4.01 (±0.41)	0.974^a^	1.01 (±0.22)	0.521^b^	10.52 (±1.20)	0.416^a^	14.02 (±1.14)	0.343^a^	25411.1 (±7484.4)	0.521^b^	25.36 (±7.05)	0.865^b^
	CSWSFR	31	4.13 (±0.39)		1.09 (±0.22)		9.91 (±1.04)		14.65 (±1.29)		20571.8 (±5626.1)		25.96 (±6.59)	
CPI–UWSFR	Code 0	18	3.93 (±2.37)	0.537^c^	1.15 (±1.47)	0.440^c^	9.28 (± 7.93)	0.076^c^	14.65 (±6.63)	0.654^d^	27613.4 (±47081.4)	0.504^c^	30.42 (±48.44)	0.302^c^
	Code 1	9	4.91 (±2.35)		0.51 (±0.30)		12.70 (±3.14)		13.77 (±3.35)		28610.2 (±40045.3)		18.40 (±25.20)	
CPI–CSWSFR	Code 0	18	4.16 (±1.83)	0.973^d^	1.20 (±1.40)	0.471^c^	9.15 (±4.11)	0.520^c^	14.35 (±5.69)	0.793^d^	21171.3 (±32099.0)	0.537^c^	33.80 (±44.81)	0.134^c^
	Code 1	9	4.20 (±2.82)		0.51 (±0.43)		11.95 (±8.08)		13.88 (±3.30)		27196.8 (±35845.0)		14.72 (± 21.12)	
CAL–UWSFR	Without CAL	9	3.78 (±2.30)	0.473^d^	0.91 (± 0.99)	0.820^c^	8.70 (±5.00)	0.366^d^	15.54 (±6.92)	0.455^d^	43225.8 (±59887.5)	0.403^c^	11.85 (±5.75)	0.631^c^
	With CAL	18	4.49 (±2.43)		0.95 (±1.37)		11.28 (±7.58)		13.76 (±5.08)		20305.6 (±33143.2)		33.70 (±50.12)	
CAL–CSWSFR	Without CAL	9	4.45 (± 1.81)	0.640^d^	1.17 (±1.33)	0.403^c^	10.03 (±3.90)	0.495^c^	15.35 (±5.35)	0.348^c^	28768.9 (±37436.4)	0.860^c^	18.71 (±27.39)	0.527^c^
	With CAL	18	4.03 (±2.34)		0.87 (±1.16)		10.11 (±6.57)		13.61 (±4.80)		20385.3 (±31039.8)		31.80 (±43.96)	
PS–UWSFR	CGHI	8	3.86 (±2.44)	0.755^e^	1.01 (±1.01)	0.716^f^	8.35 (±5.23)	0.798^e^	15.23 (±7.33)	0.944^e^	48504.7 (±61743.4)	0.264^f^	12.96 (±5.02)	0.099^f^
	GIP	1	3.20		0.15		11.50		18.00		995.00		3.00	
	GRP	8	5.12 (±2.42)		0.56 (±0.28)		12.85 (±3.32)		13.25 (±3.16)		32062.1 (±41354.0)		20.32 (±26.22)	
	RP	10	3.99 (±2.44)		1.27 (±1.80)		10.03 (±9.81)		14.18 (±6.37)		10900.5 (±22883.0)		44.40 (±62.65)	
PS–CSWSFR	CGHI	8	4.21 (±1.78)	0.582^e^	1.27 (±1.38)	0.223^f^	9.78 (±4.10)	0.713^f^	15.15 (±5.68)	0.972^e^	32262.0 (±38421.3)	0.318^f^	20.80 (±28.51)	0.062^f^
	GIP	1	6.40		0.32		12.00		17.00		824.00		2.00	
	GRP	8	3.92 (±2.88)		0.54 (±0.45)		11.94 (±8.64)		13.50 (±3.30)		30493.4 (±36832.6)		16.31 (±21.99)	
	RP	10	4.12 (±1.96)		1.14 (±1.49)		8.65 (±4.26)		13.71 (±5.91)		12298.8 (±24520.3)		44.20 (±53.75)	

Abbreviations: CAL, clinical attachment loss; CGHIP, clinical gingival health on an intact periodontium; CPI code 0, healthy periodontium; CPI code 1, gingival bleeding; CPI, community periodontal index; CSWSFR, chewing‐stimulated whole saliva flow rate; GB, gingival bleeding; GIP, gingivitis on an intact periodontium; GRP, gingivitis on a reduced periodontium; HP, health periodontium; PS, periodontal status; RP, reduced periodontium; UWSFR, unstimulated whole saliva flow rate.

^a^ Paired *T*‐test.

^b^ Wilcoxon signed‐rank test.

^c^ Mann–Whitney test.

^d^ Independent *T*‐test.

^e^ ANOVA test.

^f^ Kruskall–Wallis test.

### 3.4 Oral Health Index Comparison

Tongue coating was also more common in the NF1 group (43.0% vs. 32.6%; *p* = 0.022; paired *t*‐tests). No significant differences were noted in oral hygiene index (excluding four NF1 individuals with orthodontic appliances), salivary pH, or buffering capacity (Table 4).

### 3.5 Correlation of Periodontal Status With Salivary and Oral Health Parameters in NF1

The analysis revealed that NF1 individuals with gingival inflammation demonstrated higher oral hygiene index scores (indicating poor hygiene) than those with reduced periodontium without gingivitis (*p* = 0.035; Table 4), and also showed a higher prevalence of tongue coating in individuals with GRP compared with those with clinical gingival health (*p* = 0.007, Table 4). Curiously, oral dryness questionnaire scores were lower in those NF1 individuals with gingival inflammation (*p* = 0.005, Mann–Whitney test; *p* = 0.04, Kruskal–Wallis test). However, no significant differences were observed in salivary flow rates (resting or stimulated), salivary pH (resting or stimulated), buffering capacity (Table 4), or biochemical salivary levels across the periodontal classifications (Table 5).

### 3.6 Socioeconomic Status and Periodontal Health in NF1

Socioeconomic variables, including educational attainment and family income, showed no significant association with CPI, CAL, or overall periodontal diagnosis among NF1 participants (Table S1).

## 4 Discussion

Research into oral manifestations in individuals with NF1 has become a growing focus in the scientific literature; however, scant information is available regarding the periodontal condition and salivary function in these individuals, corroborating the need for this research. This is a pioneer study in the investigation of periodontal status and its correlation with salivary dysfunction in individuals with NF1, reporting an increased prevalence of periodontal diseases, including gingivitis and clinical attachment loss, and higher rates of salivary dysfunction, resulting in hyposalivation and xerostomia in NF1 individuals.

According to Brazil's 2023 National Oral Health Survey using CPI and CAL indexes, gingival bleeding among adults aged 35–44 (the mean age of our sample) was 41.53%, and periodontal pockets (4–5 mm) affected 13.36%. In the Southeast region, these rates were slightly higher (43.06% and 14.72%, respectively). (Ministério *da Saúde* [Brazil] 2025) Our NF1 group showed lower gingival bleeding (39.5%) but significantly higher clinical attachment loss (68.4%), more than five times the general population.

A Finnish cohort reported a hazard ratio of 3.63 for gingivitis and periodontitis in NF1 and more hospital visits for periodontitis than controls. (Reinhold et al. 2024) Thota et al. (2022) also found increased periodontal disease in NF1, mainly in individuals over 20. (Thota et al. 2022) Similarly, Bardellini et al. (2011) found no significant periodontal alterations in younger patients (Bardellini et al. 2011), suggesting an age‐dependent progression. Our adult cohort supports this trend.

The CPI and CAL indexes, which are recommended for epidemiological comparisons, were used. However, general scoring may obscure the true extent of periodontal involvement. Therefore, we assessed localized and generalized status per the new classification. Among 210 NF1 and 233 control sextants, gingivitis occurred more frequently in NF1 (35.7% vs. 9.5%). Gingivitis and reduced periodontium were also significantly more generalized in NF1, reinforcing their poorer periodontal status. Gingivitis was predominantly observed in the premolar and molar regions, whereas CAL showed a more generalized distribution. The frequency and severity of gingivitis tend to be higher in posterior teeth due to anatomical features, which include more complex occlusal and proximal surfaces. These features hinder efficient cleaning and promote greater biofilm accumulation, leading to increased inflammation (Sreenivasan and Prasad 2017).

Periodontal disease results from microbial biofilm–induced inflammation that, when uncontrolled, leads to connective tissue and bone loss. In NF1, factors such as reduced salivary flow, impaired oral hygiene, and possible microbial alterations may contribute (Giannobile et al. 2009; Hernández‐Castañeda et al. 2015; Martínez‐García and Hernández‐Lemus 2021). Socioeconomic factors like education and income showed no significant association with periodontal status; in fact, NF1 participants had higher averages vs. national data, indicating intrinsic or biological mechanisms likely underlie the observed outcomes (*Ministério da Saúde* [Brazil], 2025).

To assess whether hyposalivation influences periodontal status, we compared salivary flow with CPI and CAL within the NF1 group. Although flow rates were lower in NF1 than in controls, no correlation with periodontal measures was found. Salivary pH and buffering capacity also showed no significant differences. Thus, while hyposalivation is common in NF1, it may not directly affect periodontal outcomes. Thota et al. (2022) noted declining flow and rising pH with age in NF1, (Thota et al. 2022) suggesting salivary dysfunction may progress over time.

Since hyposalivation can promote biofilm and tongue coating, we examined their association with periodontal health (Hernández‐Castañeda et al. 2015; Martínez‐García and Hernández‐Lemus 2021; Aliyev et al. 2021). Poorer hygiene correlated with the presence of gingivitis in NF1, and greater tongue coating was also observed in cases with generalized periodontal damage. Bardellini et al. (2011) reported poor hygiene in NF1 children, though their cohort was younger (Bardellini et al. 2011).

Thicker tongue coating in our NF1 participants was linked to bleeding, attachment loss, or both. This coating harbors gram‐negative bacteria, *Candida albicans* , and Lactobacillus spp., increasing infection risk (Galimanas et al. 2014; Li et al. 2021). In hyposalivation, higher viscosity may further favor pathogenic colonization (Galimanas et al. 2014). This may explain the association between coating and periodontal status in NF1.

Studies associate elevated salivary calcium with periodontitis due to plaque calcification facilitated by alkaline pH and bacterial enzymes(Sewón et al. 1988; Sewón and Mäkelä 1990; Fiyaz et al. 2013; Rajesh et al. 2015; Romano et al. 2022; D'souza et al. 2023). We compared salivary calcium, phosphorus, and magnesium levels with periodontal indices but found no statistically significant associations. Nonetheless, individuals with worse outcomes showed slightly elevated calcium and phosphorus, possibly suggesting a trend. Salivary enzymes (amylase and lipase) showed no group differences and were not further analyzed. More research is needed.

Transcriptomic analyses have begun revealing molecular links among NF1, salivary dysfunction, and periodontal disease. Thota et al. (2022) found overlapping fibrotic signaling pathways in NF1, periodontitis, and Sjögren's syndrome. TNFSF13B was upregulated in NF1 and periodontitis, and PML and STAT1 in both NF1 and Sjögren's. These genes may be modulated by NF1 allelic loss (Thota et al. 2022). Although this study included a control group, the lack of paired statistical analysis constitutes a methodological limitation. While preliminary, these findings suggest shared pathophysiological mechanisms that merit further study.

This study has limitations. The cross‐sectional design precludes causality. Despite age and sex matching, sample size may restrict generalizability. This study was also unable to assess the causality of periodontal disease in individuals with NF1, as well as their history of previous periodontal treatment at the sites evaluated. The lack of microbiological data also limits insights into microbial contributors. Still, this study provides novel evidence of high periodontal disease and salivary dysfunction prevalence in NF1, supporting the need for integrated dental care and further longitudinal and molecular research.

## 5 Conclusion

Individuals with NF1 had poor periodontal status, higher rates of hyposalivation and xerostomia, and thicker and more extensive tongue coating vs. matched controls. Periodontal status was not significantly associated with salivary flow rates, pH, buffering capacity, salivary biochemical composition, or socioeconomic indicators such as educational level and familial income.

## Author Contributions

**Eloá Borges Luna:** investigation, methodology, writing – review and editing, writing – original draft, formal analysis. **Gustavo de Souza Vieira:** methodology, writing – review and editing, formal analysis. **Flávia Lima Kleinsorgen Motta:** investigation, methodology, writing – original draft, writing – review and editing, formal analysis. **Ana Beatriz Oliveira Gambôa:** formal analysis, writing – review and editing, data curation. **Analúcia Rampazzo Xavier:** conceptualization, writing – original draft, methodology, formal analysis, writing – review and editing, data curation. **Rafaela Elvira Rozza‐de‐Menezes:** conceptualization, writing – original draft, writing – review and editing, methodology, formal analysis, investigation, data curation, project administration. **Karin Soares Cunha:** conceptualization, writing – original draft, investigation, methodology, writing – review and editing, formal analysis, supervision, data curation.

## Funding

The authors have nothing to report.

## Consent

All patients provided written consent to participate, and this study was approved by the Federal Fluminense University Ethics Committee (#31640/2012).

## Conflicts of Interest

The authors declare no conflicts of interest.

## Supporting information


**Table S1:** Cross‐tabulation of the periodontal indexes with educational level and familial income in the individuals with Neurofibromatosis type 1.

## Data Availability

The data that support the findings of this study are available from the corresponding author upon reasonable request.
