# Supplementary material for: Periodontal Disease and Salivary Gland Dysfunction in Neurofibromatosis Type 1: A Case–Control Study
Source: Oral Dis. 2026 Apr 6;32(8):2492–502. doi: 10.1111/odi.70306 (PMC13693624; doi:10.1111/odi.70306)
Supplement: Supplementary file 1 [file ODI-32-2492-s001.docx]

**Supplementary Table 1** **–** Cross-tabulation of the periodontal indexes with educational level and familial income in the individuals with Neurofibromatosis type 1

| SCHOLARITY – NF1 | | | | | | | | | | | | | | | |
| --- | --- | --- | --- | --- | --- | --- | --- | --- | --- | --- | --- | --- | --- | --- | --- |
|  |  | | **COMMUNITY PERIODONTAL INDEX** | | | | | | | | | | | | **p-value^1^** |
| High school completed |  | | **Code 1** | | | | **Code 0** | | | **Total** | | | | | 0.979 |
|  | **Yes** | | 13 (34.2%) | | | | 20 (52.6%) | | | 33 (86.8%) | | | | |  |
|  | **No** | | 2 (5.3%) | | | | 3 (7.9%) | | | 5 (13.2%) | | | | |  |
|  | **Total** | | 15 (39.5%) | | | | 23 (60.5%) | | | 38 (100%) | | | | |  |
|  |  | | **CLINICAL ATTACHMENT LOSS** | | | | | | | | | | | | **p-value^1^** |
| High school completed |  | | **With CAL** | | | | **Without CAL** | | | **Total** | | | | | 0.103 |
|  | **Yes** | | 21 (55.3%) | | | | 12 (31.6%) | | | 33 (86.8%) | | | | |  |
|  | **No** | | 5 (13.2%) | | | | 0 (0.0%) | | | 5 (13.2%) | | | | |  |
|  | **Total** | | 26 (68.4%) | | | | 12 (31.6%) | | | 38 (100%) | | | | |  |
|  |  | | **PERIODONTAL STATUS** | | | | | | | | | | | | **p-value^1^** |
|  |  | **CGHIP** | | | **GIP** | | | **GRP** | | | **RP** | | **Total** | | 0.393 |
| High school completed | **Yes** | 10 (26.3%) | | | 2 (5.3%) | | | 11 (28.9%) | | | 10 (26.3%) | | 33 (86.8%) | |  |
|  | **No** | 0 (0%) | | | 0 (0%) | | | 2 (5.3%) | | | 3 (7.9%) | | 5 (13.2%) | |  |
|  | **Total** | 10 (26.3%) | | | 2 (5.3%) | | | 13 (34.2%) | | | 13 (34.2%) | | 38 (100%) | |  |
| MONTHLY FAMILIAL INCOME (according to Brazilian minimun wage) – NF1 | | | | | | | | | | | | | | | |
|  | | | | **COMMUNITY PERIODONTAL INDEX** | | | | | | | | | | **p-value^2^** | |
| Montlhy minimun wages | | | | **Code 1** | | | | | **Code 0** | | | | | 0.952 | |
|  |  |  |  | 3.33 (±1.44) | | | | | 4.70 (±4.70) | | | | |  |  |
|  | | | | **CLINICAL ATTACHMENT LOSS** | | | | | | | | | | **p-value^2^** | |
| Montlhy minimun wages | | | | **With CAL** | | | | | **Without CAL** | | | | | 0.195 | |
|  |  |  |  | 3.69 (±3.73) | | | | | 5.17 (±3.88) | | | | |  |  |
|  | | | | **PERIODONTAL STATUS** | | | | | | | | | | **p-value^3^** | |
| Montlhy minimun wages | | | | **CGHIP** | | **GIP** | | | **GRP** | | | **RP** | | 0.218 | |
|  |  |  |  | 5.70 (±4.05) | | 2.50 (±0.70) | | | 3.46 (±1.50) | | | 3.92 (±5.17) | |  |  |

**Legend:** CPI: Community Periodontal Index; CPI code 0: healthy periodontium; CPI code 1: gingival bleeding; CAL: clinical attachment loss; HP: Health periodontium; GB: Gingival bleeding; CGHIP: Clinical gingival health on an intact periodontium; GIP: Gingivitis on an intact periodontium; GRP: Gingivitis on a reduced periodontium; RP: Reduced periodontium; ^1^Chi-square test; ^2^Mann-Whitney test; ^3^Kruskal Wallis test.
